# Supplementary figures and images for: Characterisation and microbial community analysis of lipid utilising microorganisms for biogas formation
Source: PLoS One. 2019 Nov 8;14(11):e0224989. doi: 10.1371/journal.pone.0224989 (PMC6839884; doi:10.1371/journal.pone.0224989)

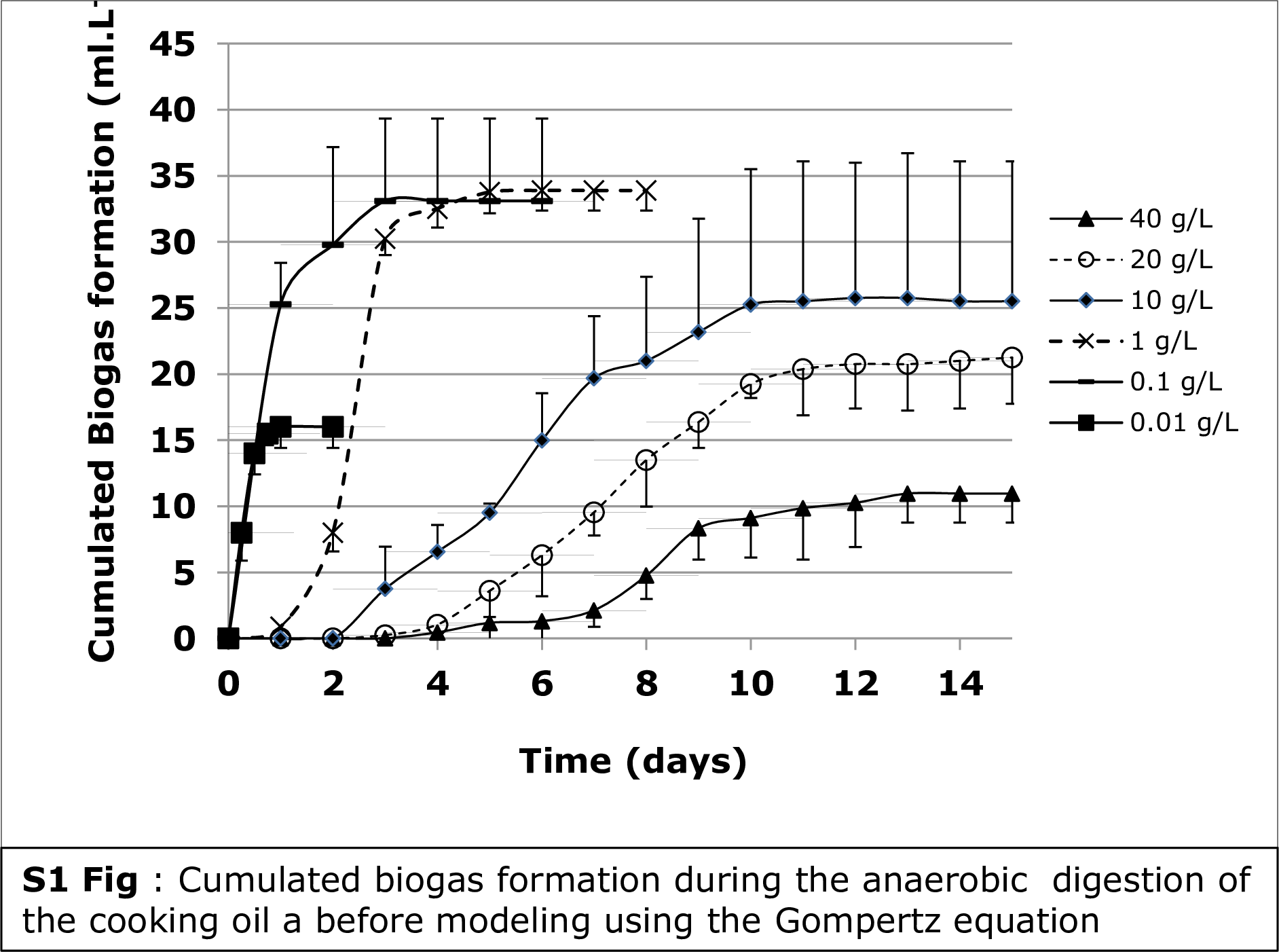

Supplement: S1 Fig — Cumulated volume of biogas formation during the anaerobic digestion of cooking oil (CO), before using the Gompertz equation model. (TIF) [file pone.0224989.s001.tif]

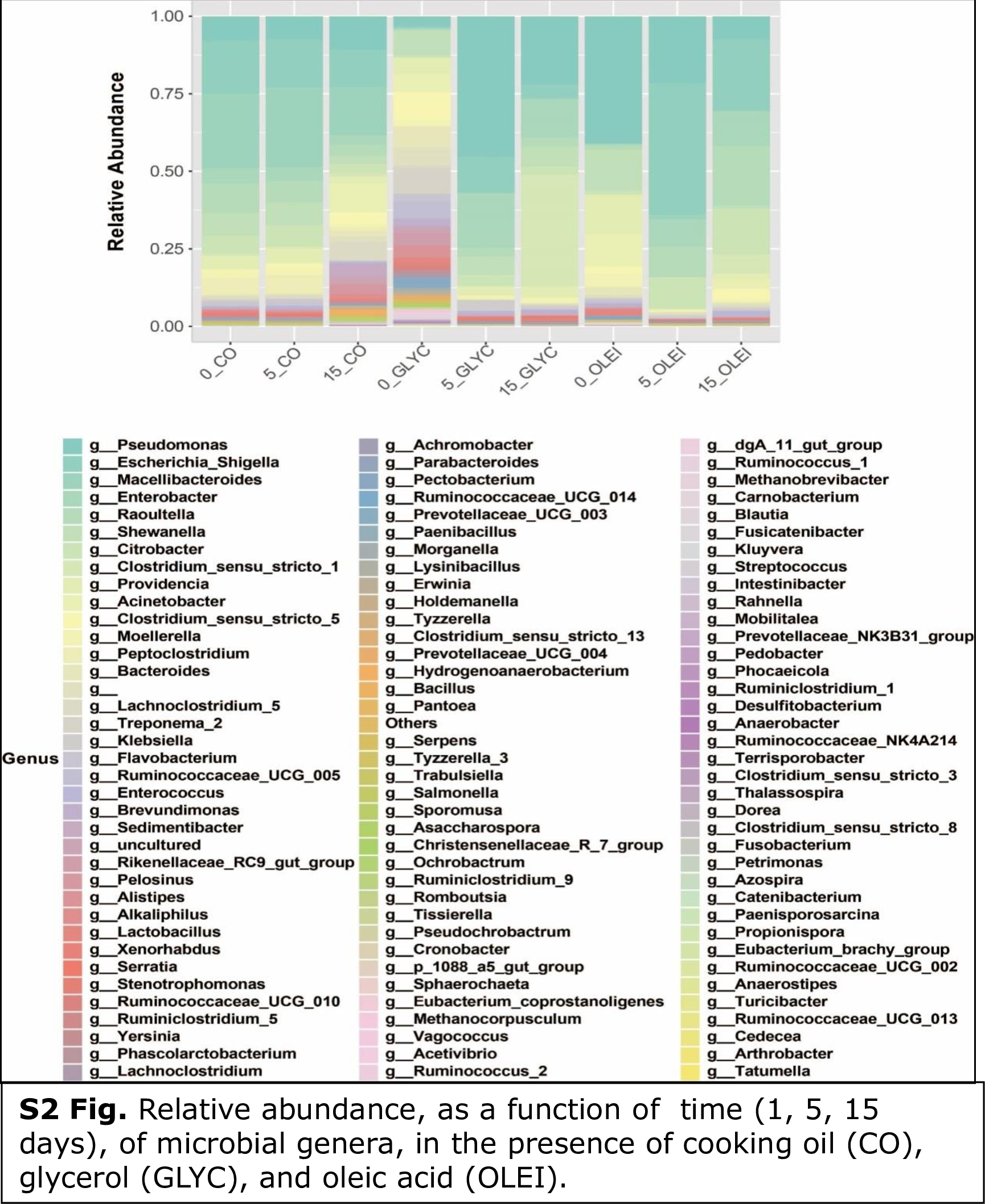

Supplement: S2 Fig — Relative abundance, as a function of time (1, 5, 15 days), of microbial genera, in the presence of cooking oil (CO), glycerol (GLYC), and oleic acid (OLEI). (TIF) [file pone.0224989.s002.tif]
