# Supplementary material for: Characterisation and microbial community analysis of lipid utilising microorganisms for biogas formation
Source: PLoS One. 2019 Nov 8;14(11):e0224989. doi: 10.1371/journal.pone.0224989 (PMC6839884; doi:10.1371/journal.pone.0224989)
Supplement: Attachment — Submitted filename: Response to Reviewer _PLOS ONE.docx [file pone.0224989.s004.docx]

RESPONSE TO REVIEWER COMMENT, PONE-D-19-1575 27 Jun at 16:42

**Journal Requirements:**

**1JR**. When submitting your revision, we need you to address these additional requirements.

Please ensure that your manuscript meets PLOS ONE's style requirements, including those for file naming. The PLOS ONE style templates can be found at
http://www.journals.plos.org/plosone/s/file?id=wjVg/PLOSOne_formatting_sample_main_body.pdf and <http://www.journals.plos.org/plosone/s/file?id=ba62/PLOSOne_formatting_sample_title_authors_affiliations.pdf>

***Our answer:*** *We have carefully read the requirements and have made the necessary changes so that our manuscript meet the journal requirements. We have also checked all the figures in “PACE application.*

**2JR**. In your Data Availability statement, you have not specified where the minimal data set underlying the results described in your manuscript can be found. PLOS defines a study's minimal data set as the underlying data used to reach the conclusions drawn in the manuscript and any additional data required to replicate the reported study findings in their entirety. All PLOS journals require that the minimal data set be made fully available. For more information about our data policy, please see http://journals.plos.org/plosone/s/data-availability.

Upon re-submitting your revised manuscript, please upload your study’s minimal underlying data set as either Supporting Information files or to a stable, public repository and include the relevant URLs, DOIs, or accession numbers within your revised cover letter. For a list of acceptable repositories, please see http://journals.plos.org/plosone/s/data-availability#loc-recommended-repositories. Any potentially identifying patient information must be fully anonymized.

We will update your Data Availability statement to reflect the information you provide in your cover letter.

***Our response:*** *We have made our data available through the addition of a Supporting information as S3_Fig_methanogenic data_excell, and the sequencing data have been made available through the NCBI site, under the following references: SUB5945674 (SubmissionID), PRJNA554546 (BioProject ID), the BioProject database (*[*http://www.ncbi.nlm.nih.gov/bioproject/554546*](http://www.ncbi.nlm.nih.gov/bioproject/554546)*)*

**Comments to the Author**

**1Co_Au**. Is the manuscript technically sound, and do the data support the conclusions?

*The manuscript must describe a technically sound piece of scientific research with data that supports the conclusions. Experiments must have been conducted rigorously, with appropriate controls, replication, and sample sizes. The conclusions must be drawn appropriately based on the data presented.* 

Reviewer #1: Partly

***Our Response:*** *We feel that our data do support the conclusion our work, and the protocols and methodology we used have been reported elsewhere and we have quoted the appropriate references.*

**2Co-Au**. Has the statistical analysis been performed appropriately and rigorously? 

Reviewer #1: No

***Our response:*** *We have included a statistician as a co-author, Dr Marwan Al-Momani, and he has re-analysed our data appropriately. All the changes are highlighted in yellow in the text. Thus, all the statistical tests are now sound and appropriate.*

**3Co_Au**. Have the authors made all data underlying the findings in their manuscript fully available?

The [PLOS Data policy](http://www.plosone.org/static/policies.action#sharing) requires authors to make all data underlying the findings described in their manuscript fully available without restriction, with rare exception (please refer to the Data Availability Statement in the manuscript PDF file). The data should be provided as part of the manuscript or its supporting information, or deposited to a public repository. For example, in addition to summary statistics, the data points behind means, medians and variance measures should be available. If there are restrictions on publicly sharing data—e.g. participant privacy or use of data from a third party—those must be specified.

Reviewer #1: No

***Our response:*** *This has been addressed, as mentioned in our response in* ***2JR***

**4Co_Au**. Is the manuscript presented in an intelligible fashion and written in standard English?

*PLOS ONE does not copyedit accepted manuscripts, so the language in submitted articles must be clear, correct, and unambiguous. Any typographical or grammatical errors should be corrected at revision, so please note any specific errors here.*

Reviewer #1: No

***Our response:*** *We have carefully re-read the manuscript have made the necessary changes.*

**5. Review Comments to the Author**

*Please use the space provided to explain your answers to the questions above. You may also include additional comments for the author, including concerns about dual publication, research ethics, or publication ethics. (Please upload your review as an attachment if it exceeds 20,000 characters)*

Reviewer #1: Review on the manuscript entitled „Characterisation and metagenomic analysis of lipid utilising microorganisms for biogas formation“ submitted to PLOS ONE (PONE-D-19-15756).
The manuscript describes anaerobic digestion experiments with fat-oil-grease as substrate, in more detail with cooking olive oil, three different long chain fatty acids and glycerol. Inhibitory of the substrates were evaluated in batch tests and the microbial communities inherent of this processes were analyzed. In contrast to glycerol which showed no inhibitory effect, the long chain fatty acids were inhibiting the biogas production even stronger than cooking oil. This was also reflecting in the different microbial community compositions.Even though the experiments are thoroughly conducted and probably scientifically sound, I would like to raise major concerns about the way data was analyzed and presented in the manuscript.

**1Re.** There was no metagenomics done! This is misleading. Metagenomics means the sequencing and analysis of all DNA present in a community. In this manuscript actually amplicon sequencing was described, so the sequencing and analysis of a single marker gene. This needs to be corrected.

***Our answer:*** *We thank the reviewer for this comment. We agree that the use of the term Metagenomics is misleading and may confuse the readers. Thus, in the text, we have changed metagenomics by “16S rRNA gene amplicon sequencing” or microbial community analysis*

**2 Re**. The methods are described to insufficient detail. I would like to highlight this with a few examples:

***Our response:*** *We have added additional information, whenever applicable, in the Material & Methods. Specific points raised by the reviewer have been addressed below.*

**a. L128-139**: Number of replicates was not given.

***Our response:*** *Experiments were carried out in duplicate and this has added in* ***line 144***

**b. L152-160:** What was measured? It is simply not mentioned here.

***Our response:*** *we thank the reviewer for this comment. Methane was measured in this experiment, and this has now been added in the text* ***line 158****.*

**c. L172:** With one-way ANOVA it can only be revealed if significant differences are present in the dataset at all. To see which ones are significantly different post hoc tests have to be applied taking into account the inflation of alpha error when doing multiple comparisons. This is not mentioned is the methods section but in Table 1 it is stated which ones are different. This can simply not be deduced from the ANOVA as described in the methods section.

***Our response:*** *As mentioned in our Response 2 Co-Au, we have included a statistician as a co-author, Dr Marwan Al-Momani, and he has re-analysed our data appropriately. Basically the ANOVA test has been combined with pairwise comparison, using TUKEY’s test, which is in line with the reviewer comment*

**d. L182:** Which primers have been used? Giving the variable regions which have been sequenced is not enough.

***Our answer:*** *We used the V4-V5 region of the 16S rRNA gene using the forward sequence 515F : 5’ - GTGCCAGCMGCCGCGGTAA – 3’ and the reverse sequence 907R-MM : 5’ - CCGYCAATTCMTTTRAGTTT – 3’*

**e. L214:** goodness of fit is not given but highly desired to judge the suitability of the Gompertz equation to fit the data presented in this manuscript.

***Our response****: To address this reviewer comment on similarity of experimental data versus those obtained by* Gompertz equation model*, we constructed a scatterplots of pairs of observations where the first coordinate is the observed experimental value, and the second coordinate is the corresponding Gompertz model value, at the same time. All graphs showed approximately a linear line from the origin. To confirm our findings, we fitted a simple linear regression model from the origin of the form Y=BX, and we tested the hypothesis B=1 versus B ≠1, and the hypothesis was not rejected in all cases, and this supports our plot’s findings. Thus, the Gompertz model equation reflects the experimental data, and this is why this model is commonly used in the biogas production. This information has been also captured in the text (see line 231).*

**3 Re.** As far as I am concerned and this is in line with a very important publication on this topic (Klindworth et al. https://www.ncbi.nlm.nih.gov/pubmed/22933715), there are no general primers which cover bacteria and archaea equally good. As the actual primers are not given I can just speculate, but primers used in this study seem to be optimized for bacteria and not for archaea because (i) the archaeal abundance is quite low and (ii) there is no acetoclastic methanogen present. Depending on the coverage of the primer for archaea (can be looked up in the mentioned publication most probably), archaeal reads should be excluded from the analysis as with these primers they cannot be sufficiently covered. This of course limits the scope of this manuscript.

***Our answer:*** *We thank the reviewer for this comment. To the best of our knowledge, there is no study yet combining primer sets for microbiome identification of archaeal and bacterial sequences. In the current study, we focused on identifying as many OTUs as we could, using a modified primer sequences that covers the V4-V5 region. During the modification of our primers, we opted to include as many archaeal sequences we could in the curation of the sequences*.

**4 Re.** L193: GreenGenes is quite old, last update was 2012 or 2013. Hence, it misses recently discovered or described organisms. Just as an example the important group of syntrophic acetate oxidizing bacteria is almost completely missing in this dataset. To include also latest knowledge use latest release of Silva database or the MiDAS database (http://www.midasfieldguide.org/en/download/) which was manually curated for activated sludge, anaerobic digesters and influent wastewater microbiomes. I strongly suggest re-running the analysis with one of the two mentioned databases. This might have little impact on the obtained results. But at least you can be sure to have the latest available information included. Both databases are already formatted to be readily used in Qiime.

- ***Our answer:*** *We thank the reviewer for this comments. As the reviewer has suggested, we have re-ran the analysis using the latest version of SILVA (132). We have employed SILVA databases, and we have obtained more OTUs with this SILVA software compared to the previous one, Greengenes.*

*However, when comparing sample counts, the observed counts were, overall, higher with Greengenes compared to SILVA. More specifically, the nine samples (1-3 for CO), 4-6 for GLYC and 7-9 for OLEI) produced counts of 17,368; 10,906; 12,232; 14,831; 20,192; 16,362; 17,507; 17,266; 16,360 respectively with the Greengenes , while those of the SILVA were (17,159; 16,310; 15,563; 14,928; 19,609; 16,065; 16,187; 17,335; 16,374). Thus, the higher number of observed counts generally came from using the Greengenes database (only 3 samples from SILVA gave higher OTU counts compared to Greengenes).*

*In relation with the alpha diversity, there was no change in shannon index, while an increase in diversity in the chao1 was observed for SILVA.*

*The PCoA result from the Bray-Curtis dissimilarity showed a more scattered pattern from the initial dates of sampling from SILVA compared to Greengenes.*

*The same clustering of the succeeding sampling dates were observed in both databases.*

*Therefore,* ***we wish to argue that there is no reason to change the database used in the study, since it had little impact on the result.*** *Moreover, the syntrophic acetate oxidizing bacteria did not increase when SILVA database was used.*

**5 Re.** Raw sequence data (fastq files) are not uploaded to a database (EMBL ENA or NCBI SRA) and hence, made publicly available. This is not in line with the PLOS data policy and data availability.

***Our answer:*** *As mentioned earlier (our Response in 2JR), the data has now been made available on NCBI database, and can be found on the link (*[*http://www.ncbi.nlm.nih.gov/bioproject/554546*](http://www.ncbi.nlm.nih.gov/bioproject/554546)*)*

**6 Re.** Spelling mistakes needs to be correctd. Here, I can just give a few examples:

***Our answer:*** *We have carefully checked the manuscript for any mistake and have made the necessary corrections.*

**a. L 50:** Methanomassiliicoccaceae

***Our answer:*** *Done*

**b. L52:** Methanobrevibacter

***Our answer:*** *DONE*

**c. L66:** archaea

***Our answer:*** *DONE*

**d. L67:** genera

**Our answer:** DONE

**e. L69:** syntrophy

**Our answer:** DONE

**f. L447:** Proteobacteria

***Our answer:*** *DONE*

**7 Re.** Rudimentary headlines are given: for example: L195, L196, L325 and so on. These are no proper headlines for a scientific manuscript.

***Our answer:*** *Throughout the text, we have re-written these headlines (see L 202, 203, 342, 414)*

 END DOCUMENT
