# Supplementary material for: Characterisation and microbial community analysis of lipid utilising microorganisms for biogas formation
Source: PLoS One. 2019 Nov 8;14(11):e0224989. doi: 10.1371/journal.pone.0224989 (PMC6839884; doi:10.1371/journal.pone.0224989)
Supplement: Attachment — Submitted filename: RESPONSE TO Reviewer Comment.docx [file pone.0224989.s005.docx]

**RESPONSE TO THE REVIEWER COMMENTS**

PONE-D-19-15756R1
Characterisation and microbial community analysis of lipid utilising microorganisms for biogas formation.

1. Primer names AND sequences need to be added to the methods section of the manuscript.

***1. Our response:*** *This has been done (page 7).*

2. Coverage of primers: To be very clear on this: There are no universal primers covering bacteria AND archaea satisfyingly. What is the coverage for bacteria and archaea for the primer pair used for this manuscript? To answer this either the primers have been used also in the mentioned study from Klindworth et al. (2013) and you can easily look that up or you need to re-run the analysis Anna Klindworth and colleagues did. However, this is not the point here. It is quite obvious when looking at the results that these primers are not covering the archaea well enough. Who is consuming the acetate as highly important intermediate during biogas formation? There are no acetoclastic methanogens detected and there are no syntrophic acetate oxidizers detected! Hence, it is strikingly obvious that these primers give a misleading picture of the archaeal community. Consequently, when knowing this, it is not scientifically sound to present the archaeal community composition based on these primers. Hence, archaeal reads should be filtered out and only the bacterial community should be presented. And yes, there are a lot of studies who use two separate primer sets, one for bacteria and one for archaea (either targeting archaeal 16S rRNA genes or mcrA gene for example).

***2.Our response***: *We fully agree with the reviewer that the proportion of methanogens is very low, which is unexpected since biomethane was produced during these experiments. The reviewer has suggested that we remove the data on methanogens, and concentrate on bacterial data only. While this acceptable, however, we would like to propose the following alternative that will take the reviewer’ comments into account. Since some methanogen archaeas have been detected, although be it at a very low proportion, we suggest to keep these data as they are, however, we have added* ***a caveat that highlights clearly the limitation of our result.*** *In this caveat (see page 21), we have clearly stated that the primers we used were not covering archaea sufficiently, hence the low level of archaea detection. Thus, with this information, we feel that the reviewer’ comment is fully addressed, while the same time, we are providing the readers with appropriate information showing the limitation of our results.*

3. Greengenes vs SILVA/MiDAS: I do not quite understand why the amplicon data analysis yields more counts, more OTUs when using Greengenes compared to SILVA. This does not make any sense. OTUs are constructed first and just after this step representative sequences of each OTU are taxonomically assigned by using one of the databases. Hence, OTU construction happens before the databases come into play and hence, is completely independent of the database. How then can the database influence the number of counts? And again Greengenes is outdate and should not be used anymore!
4. ***Our response:*** *As the reviewer has suggested, we have reanalysed our data using the SILVA/MiDAS software. News figures have been made using the data generated from this SILVA/MiDAS (Figures 6 to 10), and the text has been changed to reflect all these new results (highlighted in yellow, from page 17-21).*

*Overall, there is no major change on the result on microbial community and taxa, changes are minor, and have been incorporated in the text.*
